# Longitudinal data collection to follow social network and language development dynamics at preschool

**DOI:** 10.1038/s41597-022-01756-x

**Published:** 2022-12-22

**Authors:** Sicheng Dai, Hélène Bouchet, Márton Karsai, Jean-Pierre Chevrot, Eric Fleury, Aurélie Nardy

**Affiliations:** 1 Univ. Lyon, ENS de Lyon, Inria, CNRS, UCB Lyon 1, LIP UMR 5668, IXXI, F-69342 Lyon, France; 2 East China Normal Univ., School of Software Engineering, 200062 Shanghai, P.R. China; 3 Univ. Grenoble Alpes, LIDILEM, F-38000 Grenoble, France; 4 Univ. Rennes, Normandie Univ., CNRS, EthoS (Ethologie animale et humaine) UMR 6552, F-35380 Paimpont, France; 5 Department of Network and Data Science, Central European University, A-1100 Vienna, Austria; 6 Inria, F-75012 Paris, France

**Keywords:** Interdisciplinary studies, Computational science, Scientific data

## Abstract

DyLNet is a large-scale longitudinal social experiment designed to observe the relations between child socialisation and oral language learning at preschool. During three years, a complete preschool in France was followed to record proximity interactions of about 200 children and adults every 5 seconds using autonomous Radio Frequency Identification Wireless Proximity Sensors. Data was collected monthly with one week-long deployments. In parallel, survey campaigns were carried out to record the socio-demographic and language background of children and their families, and to monitor the linguistic skills of the pupils at regular intervals. From data we inferred real social interactions and distinguished inter- and intra-class interactions in different settings. We share ten weeks of cleaned, pre-processed and reconstructed interaction data recorded over a complete school year, together with two sets of survey data providing details about the pupils’ socio-demographic profile and language development level at the beginning and end of this period. Our dataset may stimulate researchers from several fields to study the simultaneous development of language and social interactions of children.

## Abstract (summary)

**Table:** 

Measurement(s)	RSSI values (proximity interactions within a preschool) • sociodemographic information about children • children language skills (vocabulary and syntax)
Technology Type(s)	RFID badges • questionnaire • language tests
Sample Characteristic - Organism	Homo sapiens
Sample Characteristic - Environment	preschool
Sample Characteristic - Location	France

## Background & Summary

The structure of social networks and their dynamics over time strongly influence language usage and change^1^. Conversely, the way in which individuals use language contributes to the way they are judged in society^2^, and therefore influences their friendship choices, modifying the structure of their social network. Despite this recognized relation, the co-evolution of dynamically changing social networks and language dynamics mitigated by social interactions is a largely unobserved phenomenon. Preschool environment provides an ideal place to observe these reciprocal influences. Children’s language changes rapidly during the preschool years due to the acquisition process^3^. Meanwhile, children integrate and adapt at school via socialization and increased opportunities to communicate with peers and with the adults in charge. Besides, contacts with many peers cause preschoolers to expand and restructure their social network^4,5^. This co-evolution process, through the interactions between language acquisition and socialization, has societal implications as it may promote or undermine academic success and linguistic skills. A virtuous circle - or a spiral of failure - between children’s sociability, oral communication and learning at school may therefore ensue.

Social inequalities are a key factor in this causal chain since, as of age 2, it has been observed that children from families of higher socio-economic status (SES) have a richer lexicon and use more complex syntax than children from lower-SES environments^6^. Children from different backgrounds do not use, to the same extent, the academic language that is encouraged at school^7,8^. The observations of these early differences, which are transmitted within the family^9^, led to numerous studies that have revealed the key-influence of the nature and quantity^10^ of speech addressed to children by their parents in the different social environments. School attendance introduces a new factor into the equation through peers influence, especially when the academic group is socially mixed^11^, or through the speech produced by the teachers^12^. The linguistic skills of a child will advance more quickly if they are a member of a peer group with a high level of language abilities. This effect has been observed across various indicators of language development: vocabulary, syntax, or narrative skills^11,13–15^.

The aim of the DyLNet project (https://dylnet.suniv-grenobsle-alpes.fr/en) is to observe and characterize the relations between child socialization and oral language learning during the preschool period by means of an innovative multidisciplinary approach that combines work in the fields of language acquisition, sociolinguistics and network science. This goal has been achieved via a large-scale longitudinal social experiment, where a complete preschool in France was followed, including children from three different grades as well as their teachers and teacher assistants. During the experiment we collected the proximity interactions of about 200 participants (circa 170 preschoolers and 30 adults in charge) in every 5 seconds using autonomous Radio Frequency Identification (RFID) Wireless Proximity Sensors, which were (for a large part) equipped with directional microphones allowing to record continuously the oral interactions of participants too. During the observation period of three years, data collection was conducted monthly with each deployment lasting one week. In parallel, survey campaigns using conventional techniques were carried out to record, on the one hand, the social, economic, cultural, educational, and language background of children’s families and, on the other hand, the individual level of language development of the children. All together, these simultaneous data collection efforts culminated in a long, large, and comprehensive children language development study conducted in the context of peer interaction.

In this publication, our goal is to share a cross-sectional sample recorded over one academic year. The data contain the initially cleaned and reconstructed temporal proximity social network data involving each anonymized participant, the socio-demographic information about the participating preschoolers, and their language test results collected in the beginning and in the end of that school year. Meanwhile, due to privacy reasons, the voice recordings of the participants cannot be shared. The shared data allow for several types of analysis potentially interesting not only for sociolinguists and researchers in child language development, but also for experts in social networks, human dynamics, behavioral science, education science or even social anthropology. It may open a cross-disciplinary approach to learn about how children language development depends on age, gender or socioeconomic background and how it co-evolves with the social interaction dynamics on the short and long temporal scales at the level of individuals and groups in a supervised (classroom) or unsupervised (playground) school setting.

## Methods

Novel digital technologies enable to follow human social interactions with an unprecedented resolution in time and space^16^. Over the last decade, these advancements led to an avalanche of experimental and data-driven studies addressing the precise observations of human interactions^17^ to explain phenomena like social tie formation or group dynamics. One exciting direction involves wearable devices as they allow for tracking dynamically human actions or proximity interactions at the individual level for large populations in various settings. Social studies using wearable technologies have been deployed in multiple settings such as schools^18,19^, conferences^20^ and hospitals^21,22^. These studies commonly relied on some already existing wireless architectures using RFID technology adjusted for the purpose of recording face-to-face interactions between people. Standards as *OpenBeacon* (https://www.openbeacon.org/) or *Open badges*^23^ were earlier deployed as centralized communication protocols for data collection about the relative distance and orientation of RFID tags distributed among people moving around in the same space. Our data collection method relies on similar but decentralised technology to record face-to-face interactions of children with the original goal to understand how their social interactions co-evolve with their language acquisition dynamics.

### Ethics and data protection

The DyLNet project data collection was carried out during three successive years between 2016 and 2020 to gather transactional, vocal, language and socio-demographic data about children and staff in a preschool in France. Acceptance of the experiment by pupils, parents, educational staff and school authorities, as well as issues of benefit-risk balance and privacy were carefully considered before and during the project.

The choice of the school was made with the involvement of the local and regional education authorities prior to the start of the project. The goal was to find a school with pupils from a variety of cultural, linguistic and socio-economic backgrounds and to obtain official permission for the experiment in the form of an agreement signed by the regional education officer and the university. Before launching data collection, meetings were organized with the parents and school staff to explain the purpose and organization of the experiment as well as the functioning of the RFID devices. We also offered parents the opportunity to meet individually with the researchers during drop-in sessions. In addition, we created a webpage dedicated to the families accessible from the website of the project. This webpage provided them with every details about the implementation of the project: goals, methods, benefits, risks, and the schedule of data collection (https://dylnet.univ-grenoblealpes.fr/fr/espace-familles).

The issue of children’s exposure to radio signals emitted by RFID devices has been carefully considered. We followed the advice of an expert on radio frequency safety who was a member of the French National Council for Public Health. She recommended that we have a Specific Absorption Rate measurement (i.e. rate of energy absorption per unit of mass by a human body) of the RFID device performed by an authorized company. The value obtained for one device was 0.0001683 W/kg under normal conditions of use in contact with the body, which is much lower than the European standard (2 W/kg). In addition, in agreement with the company that designed and manufactured the devices, we made sure that they complied with the European standards on the mechanical and physical properties of objects to be used by young children.

To protect the privacy of participants, we applied the principles of not mentioning participants’ names, precise locations and dates in the stored data ahead of analysis and dissemination of the results. The exact dates and timestamps at which data were collected and the child participants’ age were notably coded relatively to an arbitrarily defined *T0* set for the research project database. All participants were assigned anonymous numerical identifiers with association keys available only to the Principal Investigator. Start and end dates of data collection periods were approximated, and birth dates were replaced with children’s ages. Re-identification of participants thus appears to be impossible from the shared datasets.

As a prerequisite to be included in the study, parents (on behalf of their child) and school staff were asked to give a written consent for their participation, while informed that they could nevertheless opt-out from the experiment at any time. Non-participating children were offered to wear empty shells of RFID badges to minimize feelings of envy among classmates. Participants were asked to provide a second written consent to share the collected and anonymized data with the scientific community under the control of the Principal Investigator. Data presented in this paper include records of all the participants who consented to take part in the project, while data shared along with this paper contain only records about children and staff who provided both of these consents. For more details see Section Usage Notes. Since the assignment of children to a given public preschool in France is determined by the city council, the fact that our project was rolling for 3 years could not have any impact on the presence of, or avoidance by, certain pupils. However families had the choice to let their child join, or not, the research project. The acceptance rate was 80.63% (283/351) for pupils and 96.88% (62/64) for school staff over the three years of the project, and 80.18% (174/217) for children and 100% (32/32) for school staff for the academic year whose data are presented here and shared.

During the data collection, several participants left the venue temporally (e.g. being absent from school for health reasons) or withdrew from the experiment definitely (leaving the school as a result of house moving), as well as other new participants joined (e.g. eventually deciding to join the study, or entering the school following a house move). To help researchers keeping track of each individual’s presence in school and how much they were present on a given week, we share two tables. First, in the file “*IN-OUT table*” we summarize the enrollment status of each individual in each week. There are two states for an individual: ‘IN’ means she/he is enrolled in the corresponding week, ‘OUT’ otherwise. In another file called “*DURATION table*”, we present for each participant the duration (in seconds, computed based on pre-processed signals) she/he had data collected for each week of deployment at preschool. ‘NA’ is set for participants who had no data collected for the corresponding weeks. Note that it is possible for an individual in a certain week to be ‘IN’ in IN-OUT table while corresponding entry in DURATION table is ‘NA’: this indicates the individual is enrolled in our research project for this week but had been absent during the whole week of data collection. These two tables can be found in the shared repository in the folder called PRESENCEDURATION.

The whole project including experimental design, subject recruitment, data collection and processing, data handling, storing and sharing, privacy protection, and all aspects of the involvement of underage children were screened and approved by the ethics committee of INRIA (National Institute for Research in Digital Science and Technology) (favorable opinion, reference 2017-014, IRB00013144) as well as by the Data Protection Officer of the Université Grenoble Alpes (favorable opinion, reference CIL-UGA-2017-0980683).

### Data collection

We collected four different types of datasets during the DyLNet project. The main dataset focuses on the dynamical recording of social and oral interactions as transactional and vocal data. These data were collected autonomously using badges installed on children and school staff at the preschool (for details see Section Transactional data collection). Additionally, we gathered information about the school level of each child, that is being in 1st grade (about three years of age), 2nd grade (about four years of age), or 3rd grade (about five years of age). We also recorded the class in which the pupils and school staff were participating (out of the 7 classes in the preschool). Meanwhile, ground truth (GT) data were collected with the purpose of understanding how distance and relative orientation between a pair of badges influence the Received Signal Strength Indicator (RSSI) of recorded signals. GT data were also essential for training Machine Learning models to classify signal sequences as social interactions (for details see Sections Ground truth data collection and Temporal network reconstruction). In addition, the main data collection was accompanied with survey campaigns. A first type of survey consisted in asking parents to provide information about the socio-demographic, cultural, educational, and occupational background of the family and the daily out-of-school activities of participating children (for details see Section Socio-demographic survey data collection). Furthermore, a language survey using vocabulary and syntactic skills assessment methods was performed with all participating children once a year throughout the project to follow their linguistic development (for details see Section Linguistic survey data collection). During the 3-year observation period, we followed the interactions among preschoolers and school staff for one week each month (among the 10 months of the academic year). More precisely, we recorded data during five morning and four afternoon sessions each week, as traditionally schools are closed in France on Wednesday afternoons. In practice, children and staff were equipped with a wearable RFID badge every morning of deployment under the supervision of a researcher on site. Badges were then collected before lunch break and re-distributed in the early afternoon (or after nap time for the youngest preschoolers). Badges were collected again in the evenings for charging overnight. Data were extracted from the flash memory card of the badges at the end of each week of deployment. As the RFID badges were autonomous, they were worn not only in the classroom but also during play time when children were moving freely in the open-air yard of the school.

RFID devices provide several advantages to observe proximity interactions, as compared to alternative technologies such as video recordings, bluetooth or wireless network logs. Data retrieved from wireless network logs allow to identify co-location of participants, but without learning about their proximity and social dynamics within the given local service area. Bluetooth logs are capable to record proximity interactions, however with way less accuracy and higher noise as compared to RFID devices. Finally, although the direct video recording of an actual setting would give the most precise observations of human dynamics, these solutions are using fixed infrastructure in a given environment where they are installed, they are hardly scalable for larger observations, and it is difficult to infer social interactions from their recordings.

The applied RFID technology allowed precise recording of the proximity of participants and inference of their relative orientation up to a certain level. Moreover, badges in our experiments were autonomous and were recording signals through multiple sensors (RFID receivers and microphones). They stored the received signals locally, which allowed highly flexible employments of them in various settings (e.g. school rooms and school yards) without requiring the installation of additional static network equipments. This is in contrast to earlier RFID settings^23^ (https://www.openbeacon.org/), where badges were not equipped with large local storage but periodically transmitted recordings to storage servers via locally installed fixed wireless devices. On the other hand, while using non-autonomous solutions one could estimate the positions of individuals in the actual setting, the autonomous technology applied in our experiment allowed us to follow only relative positions of participants.

#### Transactional data collection

We employed a decentralised Low Power Wireless technology to collect transactional data between autonomous RFID badges. Each badge could be in two modes, whether broadcasting their own radio frequency ID or listening to signals emitted by other badges. To be more precise, each badge was associated with a unique ID and used the IEEE 802.15.4 low-rate wireless standard to communicate. The employed technology and its implementation meet the requirements of the product standard EN50566 following the basic restrictions of the European Council recommendations 1999/519/EC. Since badges in our experiment worked in a decentralised mode, in order to make sure that they shared consistent global time, they were first synchronised with a *synchroniser*, which was connected to a computer and propagated the same time reference to all badges. During data collection, badges broadcasted a ‘hello’ packet with 0 *dBm* transmission power for 384 *μs* every 5 seconds. For communication, they used the carrier-sense multiple access (CSMA) protocol. To avoid collision, they first listened to the dedicated channel, then transmitted a packet if the channel was clear. A badge listened to incoming packets from other devices when it was not transmitting a packet. The decentralised architecture means there was no central node to record all traffic. Instead, all badges worked autonomously and recorded locally incoming packets described by the sender badge ID, the timestamp of reception, and the RSSI. Each received signal was stored in a file locally on the flash memory card of each badge if its RSSI value overreached the minimum sensitivity value of −94 dBm. To facilitate charging of the badges (every night of deployment) and to transfer data to a local computer (at the end of every week of data collection), multi-USB hubs were designed where up to 35 badges could be plugged at the same time.

Our architecture employed three types of badges serving different purposes:

- PROX: These badges were given to participants and were hanged on their chest during data collection days. They were equipped with a battery, a memory card, and about half of them with two directional microphones for voice recordings. As soon as they were unplugged from the charging hub, these PROX badges emitted a radio signal every 5 seconds, as well as listened and recorded incoming signals on their own flash memory card. They stopped collecting data once plugged back on the charging hub.
- RX: These were special sensors, which were installed on the charging hub of each class and left unaltered during the whole week of data collection. Unlike PROX badges, RX badges only listened and recorded incoming signals (even when plugged on the charging hub). Their role was to observe when the participants belonging to a given class (attached to a given RX) were inside their classroom or not.
- FOX: This unique device was used for the time synchronization of all badges at the beginning of every week of data collection. This special *synchroniser* device was first plugged onto a PC to catch global time, then moved around the classrooms to propagate time information among all the PROX and RX badges. This device did not collect any kind of data.

At the end of each data collection period, data from each badge were transferred to a local computer. Badge IDs were then associated to the corresponding badge bearer (participant) ID within each file of contact data, and finally data were passed through an initial cleaning pipeline. The obtained cleaned data served as the input for the data pre-processing and temporal network reconstruction pipeline. All the steps of these pipelines are explained in the Section Transactional data pre-processing. We share the cleaned data along with this paper to allow researchers to build their own data pre-processing architecture.

#### Ground truth data collection

In parallel to the autonomous transactional data collection, we occasionally recorded ground truth data about actual social interactions between participants using direct visual observation methods. For two datasets (GT1 and GT2), the researcher focused on a given pair of children for a given period of observation, whereas the third (GT3) was recorded among a complete class group. Besides, while GT1 and GT3 were collected *in-situ* within a classroom with all noise and interference present (i.e. among 20–28 participants wearing a badge), GT2 was recorded in a separate room, away from other badge bearers, in controlled settings.

More specifically, GT1 consists of the recordings of the state of interaction/no-interaction between a given pair of children at a fixed 10 seconds interval (*scan sampling* method^24^), as well as their relative body orientation. GT3 corresponds to the logs at a fixed 2 minutes interval (*scan sampling* method^24^) of distances between all the children and adults of one class group during regular activities within their classroom. Both GT1 and GT3 were collected using the Animal Observer application for iPad (https://fosseyfund.github.io/AOToolBox/). These observations make possible a direct comparison between the RSSI values collected by the badges and the actual interaction state (GT1) or distance (GT3) between classmates. Finally, to get an even clearer idea of the relation between RSSI values and the actual distance between badge bearers, as well as their relative orientation, in a noise-free environment, GT2 dataset was recorded among pairs of children statically positioned at a given distance (0.1 meters, 1 meter, 2 meters) and orientation (face-to-face, side-by-side, back-to-back) for ≈10 minutes periods.

#### Socio-demographic survey data collection

Each family that consented to let their child participate in the study was asked to fill in a paper questionnaire. This questionnaire aimed at collecting information about the child and their daily family environment. In its first part, we recorded basic socio-demographic information about the participating child: gender, date of birth, birth rank and number of siblings. Other questions aimed at gathering information about the places of socialization frequented by the child before entering school (nursery, childminder…) and within the school (daycare, canteen). We also asked the parents about their child’s level of sociability, talkativeness, favorite out-of-school activities (e.g. sports, drawing, imagination/construction games…) and customary activities before sleep (e.g. story telling, cartoons, music…). Finally, two questions aimed to identify the child’s language environment and language practices at home (i.e. whether French and/or other languages are spoken within the family, and whether or not they are understood/spoken by the child).

In the second part of the questionnaire, we collected information about the child’s family environment and living place(s) (i.e. the composition of the household, and, in the case of separated families, the child’s habitual place(s) of residence). We also recorded the parents’ geographical origin, their employment status, their area of professional activity and their level of education. That questionnaire was fully completed when each child participant entered the study, then parents were asked every year to fill in a shorter version of it in order to update some information likely to change over time (e.g. the child’s favorite activities, the parents’ professional status). The shared variables are presented in the Section on Data Records later.

#### Linguistic survey data collection

Individual tests were administered to participants at school in order to assess the children’s level of language development at several points throughout the longitudinal 3-years follow-up. Tests were performed at the beginning of every school year for participants entering the study, then at the end of every school year for children already enrolled in the project. Children were evaluated individually, in a separate room, by a member of the research team. Test sessions were designed to last no more than 15 minutes, hence each child took part in two short sessions a few days apart: one during which their receptive lexical skills and short-term memory span were evaluated, and the other for assessing their receptive syntactic skills.

Language tests aimed at evaluating the participating children’s level of comprehension of words and utterances. Four versions of the tests were designed to be appropriate for each stage of preschool education, that is for pupils 1) entering 1st grade, 2) completing 1st or entering 2nd grade, 3) completing 2nd or entering 3rd grade, and 4) completing 3rd grade. The evaluation of lexical skills included 40 items (words), 30 of which being ‘test items’ specific to each version (i.e. items chosen to be adapted to the children’s school level) and the remaining 10 being ‘anchor items’ shared across all four versions (i.e. items systematically presented to the children whichever their grade, and chosen to be rather adapted to 3rd grade pupils). Similarly, the evaluation of syntactic skills contained 20 items (utterances), namely 10 ‘test items’ and 10 ‘anchor items’. In both cases, for each item, the child was presented with a plate of four pictures. The experimenter then produced the item (a word or an utterance), and the child had to point to the corresponding image with their finger.

Besides, as memory span is known to be closely linked to language skills^25^, we gave each child a memory span test as a control measure. More precisely, we asked the child to repeat after the experimenter a series of digits of increasing length. Each level contained two trials (i.e. two different series of digits of the same length). We started with a series of two digits, then proceeded with increasingly longer series (of three, four, etc.), and stopped the evaluation when the child had failed the two consecutive trials for a given level (i.e. two consecutive series of a given length). The shared variables are presented in the Section on Data Records.

### Transactional data pre-processing

Taking the raw data, we developed a data processing pipeline, as demonstrated in Fig. 2a (white rectangles), and exported data at multiple stages (indicated as blue parallelograms) of this process to share along with this paper.

#### Initial data cleaning

The data directly recorded by the badges appeared with several trivial corruptions, which were corrected during an initial cleaning pipeline. In this pipeline, we first converted the recorded binary data files to human readable format. Then we systematically removed signals that were recorded during out-of-school time, and pushed it through an individualized cleaning process. In fact, a researcher on site during each deployment week continuously followed which badge was attributed to which participant, whether a defective badge had to be replaced, whether it was dropped by a child, or if a participant was absent over a given period of time. Also, in such recorded situations, all signals emitted or received by unused, broken or missing badges were removed, not only from their own data sequence but also from the data sequence of other badges. At the end of this initial cleaning pipeline, we applied a file merging procedure to ease further analysis of the data. According to the schedule of the observed preschool, each day was divided into two periods: a morning session from 8:30 to 11:20, and an afternoon session between 13:45 and 15:50. To follow the same schedule, we merged the files of each participant into two files each day. This merging procedure allowed to retain only one file per participant per half-day, notably in cases where a participant wore different badges during that period (when a defective badge had to be replaced) or when file fragmentation had been caused by system errors. Consequently, each individual appeared with nine half-day files for each week of data collection (i.e. every mornings and afternoons from Monday to Friday, except for Wednesday afternoon) in the cleaned transactional dataset, from now on called the *cleaned data*.

#### Data pre-processing

Taking the cleaned data as input we developed a data pre-processing pipeline that we present below and whose corresponding source code we share^26^. This pipeline contains some methodological choices that we made for the purpose of our very own study. However, the shared cleaned data allow other researchers to develop their own data pre-processing pipeline with their own constraints and needs, potentially requiring other methods and parameterization than those we used. During data pre-analysis, we identified multiple issues that we corrected during this data pre-processing stage.

##### Issue 1

In the cleaned data, we identified some rare corruptions induced by a few badges: occasionally, some badges (‘silent badges’) only received signals without emitting any data packet or, on the contrary, some other badges (‘deaf badges’) only sent out signals without receiving any. To solve this issue, we copied the signal sequence that silent badges received and reversed the direction of senders and receiver, then added these reconstructed signals to the corresponding half-day files of the senders. Meanwhile, all signals received by badges that detected the deaf badge were gathered to reconstruct its incoming signal.

##### Issue 2

There were other situations where the recorded signals were meaningless, that is when badges were turned on but not worn by the participants. This happened notably when data collection was on hold and badges were retrieved and gathered before being plugged back on the charging hub. To deal with this issue, we exploited the signals received by the special RX badges which were located on the charging hub of each classroom, and thus received strong and stable RSSI from any unused badge located in the vicinity of the hub. To detect such situations, we used sliding time windows over the signal sequence of each RX badge (assigned to a given class) and computed the average and standard deviation (STD) of RSSI values for each PROX badge used in that classroom within each window. We used a relatively long time window of 3 minutes with a 1 minute step to avoid spotting situations where equipped children only approached the hub for short periods. Also, we considered only time windows with at least 80% of the expected number of signals present (i.e. at least 29 signals for a time window of 3 minutes), as otherwise it meant that the RX had temporarily lost contact with the badge (e.g. when the participant had left the classroom to get to the yard).

**Fig. 1:**
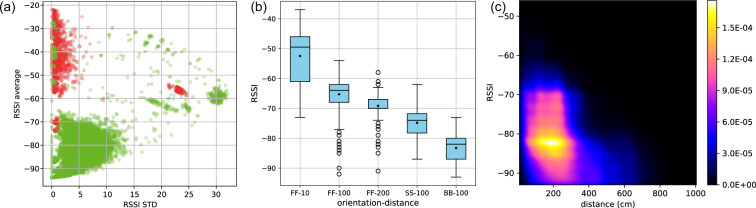
Signal strength statistics during interactions and no-interactions. (**a**) Standard deviation (x-axis) and average (y-axis) of RSSI values recorded by RX devices and coming from badges either worn (green dots) or not worn (red dots) by a child participant. Annotation is based on ground truth collected at the school (i.e. known absences). Within this uncleaned set of data, cases of misclassification are likely due to wrong manipulations. Red points in the green zone would be badges of absent children approached or manipulated by other classmates, but such cases will be solved during the Initial Data Cleaning procedure based on recorded absences. However, green points in the red zone would be badges unused or dropped by present children (notably at the beginning and end of a half-day of data collection), and these are such cases that will be filtered by the described cleaning procedure in Subsection Issue 2. Settings: window size = 3 minutes, step = 1 minute. Plot based on observational data from 7 classes, i.e. 163 children and 7 RX badges, during 1 week of data collection. (**b**) Distributions of RSSI values shown as box-plots for pairs of children observed at different experimentally-fixed distances and relative orientations (from GT2 dataset), and used to parameterize the cleaning procedure described in Subsection Issue 3. Black diamonds indicate the average value and bar is the median value. Position-distance (x-axis): letters indicate relative orientation as *‘FF’* face to face, *‘SS’* side by side, *‘BB’* back to back; and the following number indicates the distance in centimeters. Plot based on observational data from: FF-10: 1 pair, 120 seconds, 48 data points; FF-100: 5 pairs, 2765 seconds, 1106 data points; FF-200: 2 pairs, 720 seconds, 288 data points; SS-100: 1 pair, 240 seconds, 96 data points; BB-100: 1 pair, 335 seconds, 134 data points. (**c**) Correlation between distance (x-axis) and RSSI values (y-axis) shown as a density plot (from GT3 dataset). Plot based on observational data from 1 class with 28 participants, 8 observation sessions for a total of 302 minutes, 62965 data points.

To set an appropriate RSSI threshold for distinguishing between worn and unused badges, we analyzed the average and STD (i.e. the strength and (un)stability) of signals collected by RX devices and coming from unplugged active badges during the known presence or absence of the corresponding children (see Fig. 1a). Based on this analysis, we decided to set the threshold for RSSI average at −62 dBm and for STD at 2.5. These two thresholds together allowed us to achieve 93% accuracy as compared with ground truth.

To further refine data cleaning, we added two auxiliary treatments:

1. Two spotted consecutive inactive periods were concatenated if they followed each other by less than 2 minutes, i.e. if *period 1* goes from *t*_*s*1_ to *t*_*e*1_, *period 2* goes from *t*_*s*2_ to *t*_*e*2_, and *t*_*s*2_-*t*_*e*1_ < 120 seconds, then we considered as inactive the period going from *t*_*s*1_ to *t*_*e*2_. This step was necessary in order to remove isolated residual signals induced by noise between periods spotted as inactive.
2. Anytime the script identified an inactive period between *t*_*s*_ and *t*_*e*_, data were in fact also deleted from *t*_*s*_*-x* to *t*_*s*_ and from *t*_*e*_ to *t*_*e*_ + *x*, with *x* being a safety margin set at 30 seconds. This helped solving cases where badges were in the midst of being transferred from the charging hub to the participants (or vice-versa), hence recording meaningless data that the sliding time window method failed to detect because of the high STD of the RSSI values received by the RX in such situations.

These two treatments together with the sliding window method effectively removed the relatively long periods of inactivity in the cases of unworn badges before equipment of the participants or after retrieval, badges dropped on a table, or badges inadvertently lost in the school for instance.

##### Issue 3

It also happened that PROX badges could not be detected by the RX badge assigned to the corresponding class, either because the RX badge was not working properly or because a PROX badge had been dropped somewhere far from the charging hub, hence too far from the RX. To circumvent this issue and still manage to detect periods of inactivity for PROX badges, we applied a similar sliding time window method but, this time, on the signal sequence of each PROX badge. This allowed us to identify relatively long periods of inactivity when two or more PROX badges were laid together, e.g. after retrieving all the badges from a class group at the end of a day or during certain sport sessions when wearing a badge could be uncomfortable. Meanwhile, we tried to avoid deleting any signal corresponding to a potential social interaction. An actual social interaction typically involves just a pair of mutually observing badges for a short period of time and with largely varying RSSI values. To prevent spotting (and therefore deleting) such valuable series of signals, more restrictive thresholds were applied, namely −55*dBm* for average RSSI and 1.5 for STD. From the ground truth dataset collected in controlled settings (GT2), we could assess that such a high signal strength can be reached only when badges are 10 centimeters away from each other or closer, and that such a low STD is very unlikely in natural settings with badges worn by children who typically never stand still (see Fig. 1b). In natural settings, such high RSSI values are in fact almost never observed among participants during regular class activities (see Fig. 1c). Here again, we considered only time windows with at least 80% of the expected number of signals present, and we applied the two above-described auxiliary treatments (i.e. concatenation and safety margin) whenever an inactive period was spotted.

##### Issue 4

Once long inactive periods had been discarded from the signal sequence of each badge, we often observed some remaining unrealistically strong signals. Situations where badges had been gathered in close proximity during equipment or retrieval process (e.g. in a box, or in the hands of an adult) resulted in particularly strong, though unstable, signal exchanges that should not be considered as actual social contacts. Contrary to the periods of inactivity, these periods were short and with large RSSI variation. They were likely to be found at the edges (beginning and end) of each data collection period, and also occasionally within the day (e.g. when retrieving badges ahead of a sports session then equipping the children again), so sequences of signals were analyzed both forwards and backwards. During this procedure we applied a 1 minute window with a 20 seconds offset, thereby avoiding to delete longer periods because of a single strong RSSI value.

In the forwards analysis, we first spotted the first signal (with the earliest timestamp) to define the ‘initial time’ *t*_0_. Within a 1 minute window starting from *t*_0_, we scanned signals chronologically to detect signals with RSSI >−45 dBm. That threshold was chosen based on the observation that this level of signal intensity is rarely observed even when badges are 10 cm away from each other (see Fig. 1b), and actually never observed in natural settings during regular class activities (see Fig. 1c). Then:

- if none were found, this initial time *t*_0_ was kept untouched (it meant that the badge was already in use)
- if a signal with RSSI >−45 dBm was found, then its timestamp was defined as ‘strong signal time’ *t*_*s*_. The script continued to look for signals with RSSI >−45 dBm, and updated *t*_*s*_ every time a new strong signal was found within the 1 minute window.
- once we reached the end of this 1 minute window, we continued to update *t*_*s*_ only if a new strong signal was found not later than 20 seconds from the previous *t*_*s*_.

We applied this procedure the same way for the backwards analysis, but the other way round: a final 1 minute window was defined at the end of each data clip, signals with RSSI >−45 dBm were searched to define *t*_*s*_, and a step of 20 seconds was applied when the analysis continued backwards outside of the final 1 minute window.

**Fig. 2:**
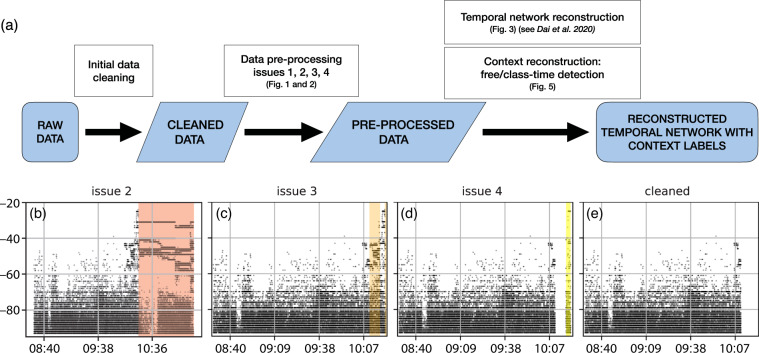
Illustration of the data processing pipeline and the issues detected during pre-processing. On panel (**a**) we show each step of data processing (white rectangles) with references to figures supporting the parameter selection at the actual processing step, and the raw (first blue rounded rectangle) and extracted (blue parallelograms) datasets shared along with this paper. Panels (**b**–**e**) demonstrate issues 2–4 and an example of the cleaned dataset. On these panels each black dot represents a signal, and color shaded areas show the problematic signals spotted by each procedure: (**b**) Issue 2 detected (red area) on a half-day file after initial data cleaning. The signals in the red area were subsequently removed; (**c**) Issue 3 detected (orange area) within the remaining signals from subplot b; (**d**) Issue 4 detected (yellow area) within the remaining signals from subplot c, and (**e**) remaining signals from subplot d constituting the final half-day file with cleaned signal sequence (i.e. pre-processed data).

These four issues were detected and treated in the order presented above. The effects of the data pre-processing pipeline are evident from Fig. 2b–e, where issues are detected step-by-step on an example of a half-day signal sequence. After solving all the issues we listed above, we obtained pre-processed data sequences for 174 children and 32 adults over 50 days during 10 weeks in the 10 months of the academic year. We used these pre-processed data as input to reconstruct the temporal network of real social interactions and to identify class-time and free-playtime periods during each day for each class.

### Temporal network reconstruction

**Fig. 3:**
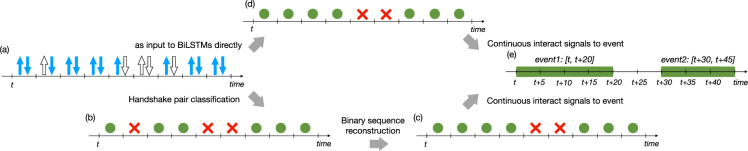
Two pipelines for temporal network reconstruction. The first path (a → b → c → e) follows a conventional way: a handshake signal sequence (**a**) is converted to a binary sequence using a classification model (step a → b), which is composed of digits that represent interaction/non-interaction states (marked as green dots and red saltires, respectively). Subsequently, (step b → c) methods like naïve, or Hidden Markov Model are applied on the binary sequences to make further corrections, resulting in a reconstructed binary sequence. Finally, from (c → d) consecutive binary interaction signals are merged to continuous interaction events (panel e). The second path (a → d → e) use the same handshake signal sequence (**a**) as input, but directly applies a Bi-directional Long Short Term Memory model, trained on the ground truth data (GT1), to provide a reconstructed signal immediately (panel d). Afterwards, the same binary event merging procedure is applied to get reconstructed events (in panel e). Note that for the same handshake signal sequence (**a**), final classified result (in panel c and d) of the two paths might be different.

To map the structure and dynamics of social interactions recorded by the RFID badges, we reconstructed them as a temporal network $G=({\mathbb{V}},\Theta )$. In this representation nodes $v\in {\mathbb{V}}$ are participants (children, school staff) and temporal edges ${\theta }_{i,j}^{t}=(t,i,j,\delta )\in \Theta$ denote interaction events between participants $i,j\in {\mathbb{V}}$ starting at time *t* and with duration *δ*. To convert signal sequences of badges to temporal social interaction events, we followed some methods we earlier developed and published in^27^. First, we identified pairs of badges, which mutually detected each other during the same period. More precisely, we took the badges *A* and *B* of two participants *i* and *j* and detected *‘handshake pairs’*, in every 5 seconds in their recorded signal sequences, as pairs of mutual observations (of the ID of badge *A* in the sequence of badge *B* and vice versa, for demonstration see Fig. 3). We matched mutual observations together if they were recorded with signals stronger than −93 dBm RSSI not further than ±2.5 seconds from each other in time. Next, taking the sequence of handshake pairs for each pair of badges, we used a logistic classification, trained on the ground truth dataset (GT1), on the RSSI values of handshake pairs to decide whether each of them corresponded to a real face-to-face interaction. Through this step, we converted the handshake pair sequence to a sequence of binary signals (as demonstrated in Fig. 3a,b). In this sequence, each binary item indicates whether there was a real interaction (coded as 1 and represented as green dot in Fig. 3b) or a non-interaction (coded as 0 and represented as red saltire) according to the classification results.

**Fig. 4:**
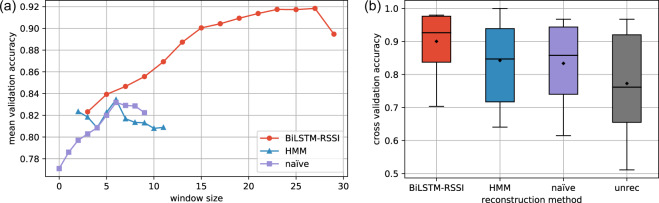
Results for the different methods of temporal network reconstruction. (**a**) Change in validation accuracy as a function of the window size of signal sequence that was used to reconstruct a single state. For naïve method this is given by the gap threshold, while for HMM and BiLSTM it is the size of the surrounding time window of a given binary state in a signal sequence. (**b**) Test set accuracy of each method shown as box-plots where black diamonds indicate the average value and bar is the median value. The BiLSTM method reaches the highest 90.03% accuracy with the smallest variation among all methods.

However, in reality more complex errors could appear in the recorded signal sequences. They may happen due to communication overload, physical obstacles, humidity or even interference with other badges. This could lead to falsely interrupted or missed interaction signals or, on the contrary, to the emergence of fake ones. Even after classification, the binary sequence could still contain misclassified signals, which could considerably alter the precise reconstruction of the real temporal interaction events. Therefore, it was essential to further conduct some corrections to the signal sequence. Using the binary signal sequence as input, we checked multiple ways to obtain a sequence of temporal events with precise starting and ending times. The simplest “naïve” event reconstruction method merges consecutive 1 signals (indicating state of interaction) into longer interaction events if they are not separated by a gap of 0 non-interaction states smaller than a given threshold (see Fig. 3b,c). Choosing a gap threshold 0 would provide a non-reconstructed sequence, while larger thresholds would filter out local noise from the signal induced by accidental packet loss. Conventionally, gap size 1 is used corresponding to 20 seconds in a typical RFID experiment^18^. This has been challenged recently by Elmer *et al*.^28^, who identified the optimal threshold being 75 seconds. Applying this “naïve” method on our data, it achieved the best 83.36% accuracy with gap size = 6, corresponding to 30 seconds. This result shows significant increased performance as compared to the direct unreconstructed outcome of the classifier (panel b in Fig. 3), which reaches 77.28% accuracy. In an earlier study^27^, we explored more complicated time-dependent methods to reconstruct interaction events from the binary signals. On the one hand, we used a Hidden Markov Model (HMM) with short-term time envelop before the actual handshake pair being reconstructed and, on the other hand, we applied a Bi-directional Long Short-Term Memory (BiLSTM) model (for details see^27^). Compared to HMM, this model includes both preceding and succeeding handshake pairs when determining the state of a given handshake pair. After training these models on 90% of ground truth data, HMM method could reach 84.25% reconstruction accuracy on the remaining unseen 10% of ground truth data used for testing, while the BiLSTM method performed the best with 90.03% accuracy (detailed results are presented in Fig. 4). Thus, we selected BiLSTM to use for the temporal network reconstruction shared along with this paper (for details see Section on Data Records).

### Free- and class-time periods

While we distinguish between morning and afternoon periods in our data collection according to the school’s schedule, we can further divide these periods into sub-periods when the children are in the classroom or in the schoolyard. These two settings allow characteristically different interaction patterns for the participants, as demonstrated in Fig. 7 by aggregating accordingly the reconstructed temporal networks^27^. During *class-time*, children are limited to interact only with peers from their own class. Moreover, they are commonly seated by the teacher in formations or around a table, in which settings potential social interactions are even further restricted. On the contrary, during *free-time*, children are free to choose to interact with anyone else from their own or any other class, as long as they are present in the schoolyard during the same free-time period. This allows for more spontaneous social interactions, and potentially larger mixing between children from different classes, grades, genders and socio-demographic groups. As we explain next, we inferred for each social interaction whether it took place during a class- or a free-time period and we share this information in the data as a feature of each temporal network event.

**Fig. 5:**
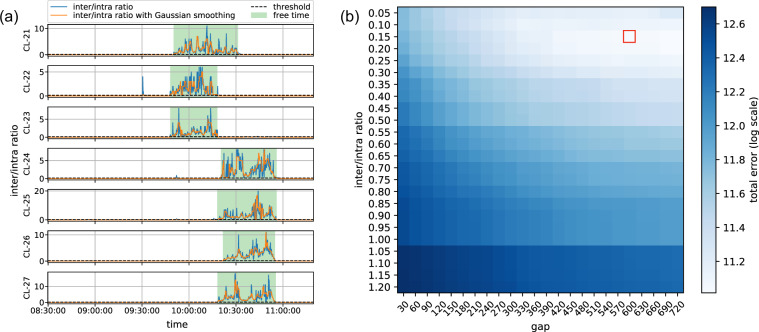
Free and class-time detection. (**a**) Examples of inter/intra ratios (as curves) for one morning period for each of the seven classes with detected free-time periods (green shaded areas) using the optimal hyperparameters. Blue curves show the original inter/intra ratios measured from the data, while orange curves depict the same curves after Gaussian smoothing (for more details see text). (**b**) Grid search of the optimal hyperparameters of gap size and inter/intra ratio minimizing classification error (coded in blue color). Optimal parameters correspond to the smallest classification error and were found at gap = 600 seconds and ratio = 0.15, respectively (marked by a red square).

To distinguish between class-time and free-time for a given class, we first counted the number of interactions that children had with peers belonging to other classes (inter-class interactions) and with peers from their own class (intra-class interactions) as a function of time. The fraction of these two counts provided the *inter/intra ratio*, which sensitively reflects whether the children were in the classroom (the ratio takes low values) or having free-time (the ratio takes larger values). Indeed, the school schedule was organized in such a way that pupils went out on free-time simultaneously with age-peers (of similar grade), thus class-groups successively met in the yard in group of 2 to 4 classes. This is demonstrated in Fig. 5a (blue curves), where the *inter/intra ratio* function is shown as curves for the seven classes in the school during a usual morning period. As can be seen on that plot, spikes appeared frequently in the observed contact numbers, which could cause false detection of free-time or class-time. To overcome this, before computing the inter/intra ratio, we smoothed the number of interactions using a Gaussian kernel with *σ* = 3 (see Fig. 5a orange curves). We then developed a method which uses a *ratio threshold* (computed in every 10 seconds) to segment every half-day into class- and free-time periods for each class. For time-steps with zero division problem, a default value was set at 2*ratio threshold. Sometimes, the ratio signal fluctuates for short periods in the middle of long free- or class-time periods. This could happen when children from one class in the schoolyard played close to a classroom where other children were taking class. Similar fluctuations appear when a class was leaving the schoolyard and another class stayed there alone for a short period of time until being joined by yet another class. To filter these short periods, we decided to bridge consecutive free-time (or class-time) periods of any given class if these periods were separated by a fluctuating period shorter than a *gap threshold*. To determine the best values of these two threshold parameters (ratio and gap), we used a grid search over an extensive range of the parameter space, as shown in Fig. 5b.

To estimate the precision of a given parameter set, we compared the identified free- or class-time periods to the ground truth data. We associated the ground truth free-time period with its start and end time *FT* = [*t*, *t*′], and duration ${\tau }_{FT}=t{\prime} -t$. Meanwhile, the corresponding inferred free-time period was between $\widehat{F}T$ = [$\widehat{t}$, $\widehat{t}{\prime}$] with duration ${\tau }_{\widehat{F}T}$. If $\widehat{F}T$ and *FT* overlapped, the overlapping period fall within ${\rm{diff}}(\widehat{F}T,FT)$ = [$max\left(t,\widehat{t}\right),min\left(t{\prime} ,\widehat{t}{\prime} \right)$] with duration denoted as *τ*_*diff*_. We defined error of free- and class-time identification as $error={\tau }_{\widehat{F}T}+{\tau }_{FT}-2{\tau }_{{\rm{diff}}}$, by comparing corresponding inferred and ground truth data from the same period. The minimum error of free- and class-time identification (computed from the recording of the actual schedule of the 7 classes on site, over a four months sample of the data for morning periods) appeared for a ratio threshold of 0.15 and a gap threshold of 600 seconds (as shown in Fig. 5b). The total error computed for all classes with these two parameter values was 60,768 seconds, which is negligible as compared to the total observation time of 1,941,000 seconds for the four-month period considered. The identified free-time periods for a typical morning session are demonstrated as shaded areas in Fig. 5a.

In most cases, this method resulted in the identification of free-time periods shared by two or more classes, who were simultaneously in the yard, as expected based on our knowledge of the school typical organization. In few occasions though, only one class was detected as being on free-time. A high *inter/intra ratio* could indeed occur when one class was on the move to the yard, children being spread out in the corridor and passing by the other classrooms, thus having numerous short contacts with pupils from other classes. During such periods where only one class is out of its classroom, interactions among children can still be considered to be restricted to peers from the same class. Thus, we applied a final correction that consisted in aligning the detected free-time periods for the first two classes out in the yard, and the last two classes leaving it, on every half-day. By doing so, we retained only free-time periods where at least two class-groups were together out in the playground, that is periods where pupils could possibly interact with peers from other classes.

## Data Records

The datasets described in this section are shared in^26^ together with the data descriptors, the generating codes, and the Data Usage Agreement that has to be completed to obtain access.

The observation period of DyLNet is spanning over three years. However, during this period, several children entered or left the school for multiple reasons (e.g. graduation, family relocation). To minimize this dynamical change of the observed population, we share data collected during a period of ten months, covering one full academic school year ranging in two civil years (from September to June). More specifically, we share five datasets: *cleaned interaction sequences, pre-processed interaction sequences, ground truth data, temporal network data*, and *socio-demographic and linguistic data*. Interaction sequences were collected each month for one week, while linguistic tests were conducted twice, at the beginning and end of the recorded school year. Finally, the answers to the socio-demographic questionnaire were collected once upon the school entry of children, and ground truth datasets were recorded occasionally during the project. The observed set of children was split in seven classes from all three grades of the preschool: classes 1 and 2 were composed of 1st grade children, class 3 was a mix of 1st and 2nd graders, class 4 consisted in 2nd graders, class 5 was a mix of 2nd and 3rd graders, and finally classes 6 and 7 consisted in 3rd grade children. All classrooms were set up in a similar way. The classroom space was divided into a group activity area (with benches), an instruction workshops area (with tables and chairs for small groups to sit around), and a free play area (with imagination and construction games, as well as story books for instance). Depending on the time of day, the pupils could be either sitting at a table or on a bench, or moving freely around the classroom or within the play area. On average, the size of a classroom was approximately 60–70 square meters.

### Interaction sequences

The sequences of recorded interactions, which underwent the initial data cleaning, are shared in standardized files, which are systematically organized in a folder structure. To store the recorded interaction sequences, for each recorded month, a root directory has been generated with the name *Y_WEEKXX* where *Y* indicates the civil year number (it takes values *Y* = 1 for the period from September to December, i.e. the first part of the recorded academic year, and *Y* = 2 for the period from January to June, i.e. the second part of the recorded school year). *XX* indicates the week number (out of 52) within each civil year. Cleaned files of interaction sequences as well as all processed and reconstructed files are stored in the same root directory system.

Each participant is identified by a unique ID called DyLNet ID (DID), which is stored as a 4-digit decimal number. The first digit of DID is 0 for children and 1 for adults (school staff), and the other three digits have been assigned to participants at random. Similarly, RX badges assigned to the different classrooms are identified by a 4-digit decimal DID, whose first digit is 2 and whose last digit is the number of the class (e.g. RX-2001 is assigned to class 1). Two additional RX badges were assigned to the two different gym rooms of the school (RX-2010 and RX-2011) in an attempt to allow for the detection of sport sessions at a later stage of the analysis.

#### Cleaned interaction sequences

Due to the DyLNet architecture, the collected interaction files were recorded locally on each badge, and then were extracted and organized into sub-folders depending on the type of badge, i.e. separated in *PROX* (participant’s data) and *RX* (RX badges’ data) subfolders. During the data recording, a researcher on site kept track of the badge assignments and recorded incidents (e.g. absence of a participant, replacement of a defective badge, or when a badge was inadvertently dropped). Data extracted from the badges could then be associated with a given participant, and recorded incidents were taken into account during the initial data cleaning procedure (as explained in Section Initial data cleaning). For each participant and each RX, the initially cleaned files were then stored in comma separated value (csv) format in a folder named *HD_individual_cleaned*, further subdivided into *PROX* and *RX* subfolders. Files were named *CID-D-DID-MA.csv* to evidently indicate the class (*CID*), day of the week (*D*: from Monday to Friday), participant (*DID*), and morning or afternoon period (*MA*) when the recording took place (for the meaning of abbreviations see Table 1). In the end, there was one cleaned data file per participant - if present at school, per half-day for each week. In these cleaned data files, the signals received by each individual from nearby participants are recorded in triplets as *(observed DID, timestamp, RSSI)*. The first item gives the *DID* of the observed participant. The second item is a *timestamp* indicating the time of observation in seconds that has passed since 12 A.M. of the day arbitrarily chosen to be used as start time *T0* for the database of the project. Finally, the third item, *RSSI*, corresponds to the received signal strength (in dBm) from the badge of the observed participant.

**Table 1:** Description of abbreviations used in file names.

*Abbreviation*	Description	Possible values
CID	Class number	1–7
D	Day of the week	1–5 (i.e. Monday-Friday)
DID	DyLNet ID of participants	4-digit decimal number
MA	Morning or afternoon observation	M/A
RSSI	Received Signal Strength Indicator	−93 dBm up to –16 dBm
RM	Reconstruction method	blstm_RSSI/unrec

#### Pre-processed interaction sequences

Next, these cleaned data files were used as input for data pre-processing. To remove recorded signals which corresponded to issues 1–4, the cleaned data files were treated (as explained in Section Data pre-processing), and the resulting pre-processed files were stored in a folder named *HD_individual_preprocessed*, which retains the structure of the folder *HD_individual_cleaned*. The format of file names and structure of data remain identical as well. Pre-processed files were later used as the input for the reconstruction process. Along with this paper, we share the cleaned data files as well as the pre-processed data files.

### Ground truth data

Several ground truth datasets have been occasionally collected by a designated researcher on site. The GT1 dataset, containing logs of interaction states and relative orientations between pairs of classmates, was collected from 7 different dyads, for a total of 3 hours 17 minutes of observations (range: 17–54 minutes per pair). The GT2 dataset was recorded between 5 pairs of children, each pair of them successively positioned in two different distance and orientation settings while holding a static position for a few minutes (range: 2–10 minutes per setting). This setting provided a total of 70 minutes of observations. Finally, the GT3 dataset, containing records of distance between all participants within a classroom, was collected among a class of 28 individuals (18–26 children and 1–2 adults wearing a PROX badge per session), during 8 observation sessions for a total of 5 hours and 2 minutes of data (average session duration: 38 minutes, range: 4–90 minutes per session).

These three datasets are shared in the folder *GROUNDTRUTH* in files named as *GT1.csv*, *GT2.csv* and *GT3.csv* along a file describing the content and coding of each variable (*GT_Variables.csv*). Note that in these ground truth datasets, participants are not referred to using the DyLNet ID(DID) described in Section Interaction sequences, but instead using upper case letters. In fact, these ground truth data have been collected to calibrate the recording system (i.e. RFID badges) ahead of the transactional data collection period, hence not involving participants that are present in the other datasets we share along with this paper.

### Reconstructed temporal network data with free/class-time annotation

As introduced in Subsection on Temporal network reconstruction, annotated ground truth dataset GT1 was used to train the model to reconstruct events based on the pre-processed data. In the shared dataset, temporal network files are named ‘*D-MA-RM.csv*’ (for the meaning of abbreviations see Table 1), where ‘RM’ = ‘blstm_RSSI’ if reconstruction method follows the second path (a → d → e) described in Fig. 3, and ‘RM’ = ‘unrec’ if the temporal network is based only on classification results without reconstruction. Temporal network files are stored in the folder *‘tnet’* under the root directory. More precisely, for each DID pair, our reconstruction model takes their dyadic signals from pre-processed files as input and reconstructs interaction events. For each given half-day, events of all DID pairs form the temporal network, which is then stored as a ‘csv’ file where each row is an interaction event ${\theta }_{i,j}^{t}=(t,i,j,\delta )\in \Theta$ as explained in Section Temporal network reconstruction. Furthermore, since we distinguished between free-time and class-time for each class-group, we annotated each event with four state labels ${S}_{t}^{i}\;{S}_{t{\prime} }^{i}\;{S}_{t}^{j}\;{S}_{t{\prime} }^{j}$ and a binary flag *X*. State labels are denoted ${S}_{\{t,t{\prime} \}}^{i}\in \{F,C\}$ where ‘*F*’ and ‘*C*’ indicate free- and class-time respectively. ${S}_{t}^{i}$ indicates whether *i* was in class-time or in free-time at time *t*, while the binary flag *X* indicates whether *i, j* are from the same class or not. For example, the event *θ* = (10, *i*, *j*, 40) and its corresponding label *FCFC*1 would refer to an interaction between two children *i* and *j*, belonging to the same class (indicated by ⋅ ⋅ ⋅ ⋅1 at the end of the label). In this case, both of them were in free-time at *t*=10 when the interaction event started, as indicated by the first and third letters (respectively *F* ⋅*F*⋅). However, they were in class at the end of the interaction event (at *t*' = *t* + *δ* = 50) as indicated by the second and fourth letters (respectively ⋅C ⋅*C*).

Over the whole year of data collection, most frequently observed types of interactions were *CCCC*1 (81.32% of the total number of interaction events, accounting for 18,465 hours of dyadic contacts) corresponding to classmates interacting during class-time, and *FFFF*1 (8.48%, i.e. 1,114 hours) corresponding to classmates interacting during free-time. Less frequent patterns were *FFFF*0 (6.85%, i.e. 755 hours) corresponding to children from different classes interacting during free-time, *CCCC*0 (2.07%, i.e. 328 hours) corresponding to children from different classes interacting during class-time (e.g. visiting each other class, meeting in the corridor or in the lavatory), and *CFCF*1 (0.35%, i.e. 134 hours) and *FCFC*1 (0.36%, i.e. 95 hours) corresponding to classmates starting an interaction in class that continued in the yard or vice-versa.

In Table 2 we summarized the total count of each type of interaction we inferred for every event in the whole dataset corresponding to one academic year (i.e. 10 weeks of data), and the total duration of dyadic interactions they represent. The table contains the following types of records:

- Type of interaction: Each event is labeled with four state tags ${S}_{t}^{i}\;{S}_{t{\prime} }^{i}\;{S}_{t}^{j}\;{S}_{t{\prime} }^{j}$ and a binary flag *X*. State labels are denoted ${S}_{\{t,t{\prime} \}}^{i}\in \left\{F,C\right\}$ where ‘*F*’ and ‘*C*’ indicate free- and class-time respectively. ${S}_{t}^{i}$ indicates whether *i* was in class-time or in free-time at time *t*, while the binary flag *X* indicates whether *i*, *j* are from the same class or not.
- Count: Number of interactions of each type, also expressed as % within the dataset.
- Duration: Total duration of each type of interaction, also expressed as % of the total time of interaction events within the dataset.

**Table 2:** Free- and class-time events annotation statistics.

Type of interaction	Count	Count (%)	Duration (sec)	Duration (%)
*CCCC1*	604,065	81.32	66,472,625	88.04
*FFFF1*	63,009	8.48	4,011,285	5.31
*FFFF0*	50,878	6.85	2,716,530	3.60
*CCCC0*	15,347	2.07	1,180,295	1.56
*FCFC1*	2,641	0.36	343,315	0.45
*CFCF1*	2,567	0.35	481,575	0.64
*FFCC0*	1,650	0.22	70,735	0.09
*CCFF0*	1,007	0.14	39,095	0.05
*FFFC0*	350	0.05	37,130	0.05
*CFFF0*	308	0.04	37,795	0.05
*FCFC0*	213	0.03	31,955	0.04
*FFCF0*	208	0.03	30,240	0.04
*FCFF0*	196	0.03	22,030	0.03
*CFCF0*	146	0.02	12,860	0.02
*FCCC0*	90	0.01	5,405	0.01
*CFCC0*	67	0.01	4,695	0.01
*CCCF0*	52	0.01	3,490	0
*CCFC0*	45	0.01	1,935	0
*CFFC0*	5	0	2,205	0

### Socio-demographic and linguistic data

We collected information from families for the 174 child participants through a socio-demographic survey. Data shared along with this paper correspond to the most up-to-date information for the year during which transactional data were collected. While some piece of information could be directly used as variables (e.g. birth rank and number of siblings), other had to be coded for either privacy reasons or analytical purposes. For instance, age is shared in months at the arbitrarily set *T0*, calculated from the recorded exact birth date that cannot be directly shared, while parents’ profession and diploma have been coded into hierarchized categories as these details could not be exposed. Besides, to ease analysis, favorite activities were grouped into a limited number of categories then coded as a yes/no value, as multiple answers to that question were frequent. Also, the coding of professions and educational level into categories facilitates further global analyses. Finally, there are some information that we collected but cannot share without compromising the anonymity of participants, which is the case of the exact nature of the languages spoken at home and the geographical origin of the parents for instance. Along with this paper, we thus share socio-demographic data about the child participants concerning only: their age in months, gender, birth rank and number of siblings, who did take care of them before entering preschool (childminder, childcare center, family members), whether they knew other pupils before entering that preschool, whether they use canteen and daycare (morning/afternoon) services, how sociable and talkative they are within their family home, which are their favorite activities out-of-school (sports, books, gaming, imagination/construction games, drawing, cartoons, board games) and right before going to sleep (cartoons, books, lullaby, playing, story, bottle-feeding, breast-feeding, music), their language environment (mono- vs. multilingual) and language practices (number of languages mastered) at home, their parents’ professional status (active/not), area of professional activity (coded in 4 categories) and their level of education (coded in 5 categories).

Two times during the observation period, linguistic surveys were carried out with each child participant. A total of 172 pupils took part in the first linguistic survey, ahead of the start of transactional data collection (that is ‘P1’ period of testing), while 169 pupils participated in the second one, at the end of data collection for that academic year (‘P2’ period of testing). Out of the 174 participants, 167 children took part in both test periods, other participants either left or entered the school over the course of the academic year. At every period of testing, two types of language tests were performed: one for lexical skills (we call ‘vocabulary’), and one for syntactic skills (we call ‘syntax’) (for details see Section Linguistic survey data collection). For each of these two tests, we computed three scores per child: one for the test items adapted to the child’s grade (out of 30 for vocabulary and 10 for syntax), one for the anchor items systematically presented irrespective of the child’s grade (out of 10 for both vocabulary and syntax), and an overall success score combining test and anchor items (out of 40 for vocabulary, out of 20 for syntax). Within each score, a successfully passed item was worth one point. Beside language tests, pupils also completed a short-term memory test. The memory span score is the level passed by the child subject, that is the length of the longest series of digits they could recall, being given two trials at each level (i.e. each length). This score thus corresponds to the highest number of digits the child could correctly repeat after the experimenter.

This dataset is shared in the folder *SOCIODEMOLING* (file *SocioDemoLing_Data.csv*), along a file describing the content and the coding of each variable (file *SocioDemoLing_Variables.csv*). A total of 61 variables are shared for each child participant: 5 collected on site (participants’ age-class, class-group, grade, and whether they took part in ‘P1’ and ‘P2’ periods of the linguistic survey), 36 collected through the socio-demographic questionnaire filled by the families of participating children, and 20 corresponding to the language test results (period, version, and scores).

## Technical Validation

### Inferred network validation

**Fig. 6:**
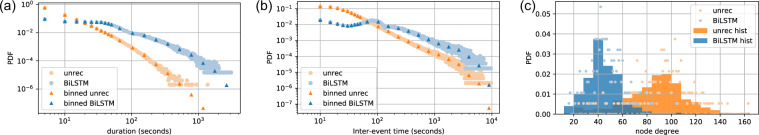
Statistical characteristics of the observed social temporal networks. (**a**) Distribution of event duration; (**b**) Distribution of inter-event times on links; (**c**) Distribution of node degrees. Dark symbols (in panels a and b) and bars (in panel c) indicate the binned distributions of the corresponding probability density functions (PDF) (light symbols). Note the logarithmic axes in panels a and b. Results are shown for the unreconstructed (orange) and the BiLSTM reconstructed (blue) structures.

To validate the structure and dynamics of the inferred social temporal network, we present here some frequently used statistics about the unreconstructed and reconstructed temporal structures. For this presentation, we randomly chose a week in the dataset and aggregated its morning periods into a sample temporal network. First, the distribution of event duration, presented in Fig. 6a, appears with fat tails for both unreconstructed and reconstructed networks, similar to earlier observations made in independent systems^29^. However, while the distribution corresponding to the unreconstructed network decreases monotonously, the distribution for the reconstructed network starts with a plateau (corresponding to the range of 5–30 seconds). Moreover, probabilities for the first three duration values (5–15 seconds) are lower for the reconstructed network than for the unreconstructed one. That reduced probability of short durations is due to the reconstruction method merging short events separated by short inter-event times into longer interactions, making low values less frequent. The reconstruction process also accounts for the different scaling of the tails of the distributions. Indeed, longer events appear with a higher probability and over a larger range in the case of the reconstructed network.

Similar differences can be observed for the inter-event time (IET) distributions measured on links, which are shown in Fig. 6b. This metric measures the length of time between the end of an event and the beginning of the next one for each pair of interacting participants. Its scaling indicates how heterogeneous an activity sequence is in time. If it appears with a broad tail, it demonstrates that the observed dynamic is bursty, thus characterized by short periods with events separated by short inter-event times, and alternating with long periods of inactivity. As it has been observed in similar systems^29^, the inter-event time distribution appears with a long tail both for the unreconstructed and reconstructed temporal networks. Similar to the duration distributions, short inter-event times become underrepresented after reconstruction as compared to the unreconstructed case. For larger values, both distributions scale close to a power-law, but with longer IETs more likely in the reconstructed temporal network. These differences can again be explained by the merging process, which commonly bridges short inter-event times between events, this way creating longer interactions and removing short inter-event times. Nevertheless, the scaling of the tails of both distributions is similar since the reconstruction model does not connect two events separated by a very large gap, thus not affecting the frequencies of long IETs.

Furthermore, to verify some structural character of the aggregated network, we measured its node degree (number of neighbors) distribution, plotted in Fig. 6c. Similar to earlier observations^29^, the degree distribution of both of the networks indicates certain heterogeneity. The unreconstructed network appears with a broader range of node degrees, ranging from 24 to 163 with an average of 90.4, while the reconstructed structure is more homogeneous with degrees between 16 and 104, for an average of 47.1. This difference is evidently caused by the reconstruction method, which filters a number of falsely observed interactions induced by noise and other external effects. Furthermore, the reconstructed network, by observing preceding and succeeding signals, gives a more accurate evaluation of whether exchanged signals between two participants indicate a real interaction or just an accidental encounter. Therefore, the number of neighbors is reduced in the reconstructed network.

**Fig. 7:**
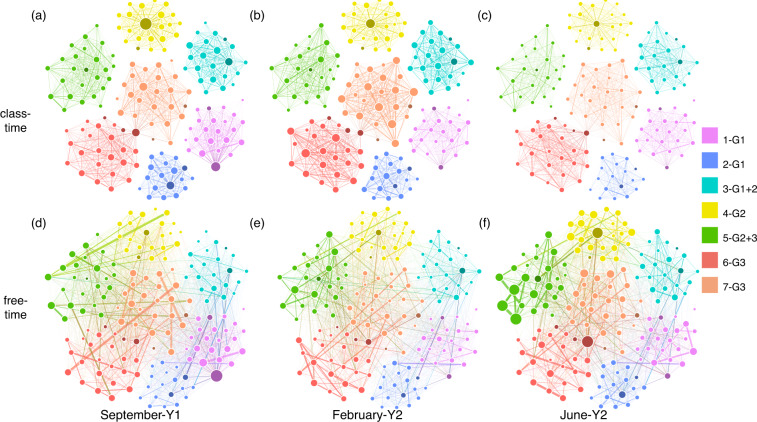
Aggregated network for morning periods over a week in September, February and June of the academic year. Networks are shown in panels (**a**–**c**) for class-time interactions, and in panels (**d**–**f**) for free-time periods. Node color indicates the class, node size scales with the total duration of interaction time for the corresponding participant, and edge width represents aggregated duration of interaction between two participants. Nodes with darker shaded colors are adult participants. In the caption, next to the class number, the class-group composition is given in terms of grade(s): G1 for 1st grade, G2 for 2nd grade, G3 for 3rd grade.

Finally, to present directly the structure and long term evolution of the observed social network, we show three successive networks in Fig. 7 during class-time (upper panels) and free-time (lower panels) periods, which were aggregated over three distinct weeks of observation to illustrate the beginning, middle, and end of the academic year (i.e. September, February and June, respectively). Here, node sizes indicate the total duration of interaction for each participant, the width of links scale with the total duration of interaction between peers, and dark shaded nodes are assigned to adult participants. Classes colored in different ways are easily identifiable from the structure, especially during class-time observations (see Fig. 7a–c). On the other hand, free-time observations (Fig. 7d–f) evidently demonstrate the different, potentially inter-class mixing patterns between children.

### Linguistic data validation

**Fig. 8:**
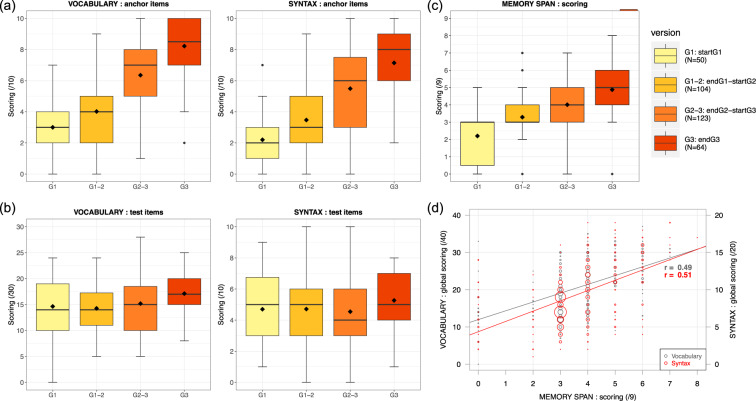
Language test results. Results are shown as box-plots, for children of different grades tested at the beginning and end of the academic year during which transactional data collection took place. Four versions of the tests were designed to be adapted to subjects’ grade (G1: *startG1* version of the tests designed for children entering 1st grade, G1-2: *endG1-startG2* version for children completing 1st grade or entering 2nd grade, G2-3: *endG2-startG3* version for children completing 2nd grade or entering 3rd grade, G3: *endG3* version for children completing 3rd grade). For both vocabulary and syntax tests, children were presented with two types of items: (**a**) anchor items, shared across versions, and (**b**) test items, adapted to the subject’s grade. (**c**) Scoring for the memory span test during which identical series of digits to repeat were used whichever the subject’s grade. In every box-plot, black diamond indicates the average value and bar shows the median value. (**d**) Correlation between memory span and performance in linguistic tasks for children of all school levels combined. Circle size is proportional to the number of data points at each (x,y) coordinates. Each of the six plots was drawn from 341 points originating from 174 children tested twice in the academic year (or only once for just 7 of them who left or entered the school over the course of the year).

In both the lexical (vocabulary) and syntactic skills (syntax) tests, we used two types of items. Ten identical so-called ‘anchor items’ were presented to children whichever their grade, every time they took a test (at every period of testing all along the project). These anchor items had been meticulously chosen to allow the researchers to evaluate linguistic skills development over time. Results shown in Fig. 8a confirm that subjects’ scores indeed increase with age. Besides, different so-called ‘test items’ were used in the four different versions of the tests adapted to the subject’s grade, namely 30 items in the vocabulary test and 10 in the syntax test. These test items had been carefully selected to allow to evaluate the level of linguistic abilities in children of similar school level. Results presented in Fig. 8b show that scores for these items are, as expected, centered around the average (≈15/30 for vocabulary, ≈5/10 for syntax). Moreover, the scores within each group (i.e. each test version) are widespread, thus meeting the requirements for achieving the goal to distinguish between high-, medium- and low-skilled pupils. These results attest to the relevance of the linguistic tests we designed specifically to achieve our objectives, which were two-fold: first to assess the improvement of linguistic skills in pupils over time, and second to measure the heterogeneity in language ability levels within same-grade groups.

In addition, the memory span test was used as a control measure, given that linguistic skills are known to be constrained by working memory capacities in developing children^25^. Children were asked to repeat the exact same series of digits whichever their grade, allowing the researchers to evaluate memory skills improvement in subjects over time. Subjects’ scores indeed increase with age (Fig. 8c). More interestingly, the fact that language test scores correlate with memory span scoring (as shown in Fig. 8d) is an additional way of validating the relevance of the linguistic tests we designed specifically to achieve our research goals.

### Studies for language-network interaction

The dataset has the potential to allow for studying the interaction between change in network structure and change in child language. First, exploring homophily phenomena based on linguistic similarity and their evolution over time is a way to study the influence of language on the network. Second, using the study of communities and interaction as a starting point, the data allows to explore the influence of the network on language, i.e., how social influences shape language skills. It should be noted that the longitudinal nature of the dataset with respect to network structure and language changes allows actual predictions to be made. If two individuals share similar linguistic level at time T, does the probability that they will interact increase at T + n? Similarly, does the frequency of interaction between two individuals at time T predict the change in their linguistic similarity at time T + n?

## Usage Notes

Note that the data presented in this paper were recorded in 174 children as study subjects, however observations of only 164 children are shared as parents of 10 children opted out to be included in data shared with researchers outside of the project team. In addition, data about 32 adult study subjects are shared. Furthermore, as the data contain sensitive information on human subjects, it cannot be shared fully openly and used freely. Access to the data can be granted by the Principal Investigator (Aurélie Nardy - corresponding author), after submission of a short research proposal, via email, on the planned use of the recorded data. Access to the data is conditional to the prior signature of the Data Access Agreement. The data can be exclusively used for scientific purposes.

## Data Availability

The program codes for data cleaning and temporal network reconstruction are shared along the dataset in an open repository. The codes have been developed in Python language using only standard or open licensed packages, and they are shared as iPython notebooks at^26^.
